# Memory circuit dosimetry in patients with brain metastases treated with Sparing Memory with Advanced Radiosurgery Targeting (SMART) technique

**DOI:** 10.1016/j.ctro.2026.101267

**Published:** 2026-08-15

**Authors:** Nina Lo, Shada Wadi-Ramahi, Hong Wang, Doris Dimitriadou, Ron Lalonde, Travis McCaw, Kanika Sarna, Christopher Wilke, Joseph Mettenburg, Kalil Abdullah, Andrew H. Zureick, Serah Choi

**Affiliations:** a Department of Radiation Oncology, University of Pittsburgh, Pittsburgh, PA, United States of America; b Department of Radiation Oncology, UPMC Hillman Cancer Center, Pittsburgh, PA, United States of America; c Biostatistics Facility, UPMC Hillman Cancer Center, Pittsburgh, PA, United States of America; d Department of Radiology, University of Pittsburgh, Pittsburgh, PA, United States of America; e Department of Neurosurgery, UPMC Shadyside Hospital, Pittsburgh, PA, United States of America; f Department of Neurological Surgery, UPMC Hillman Cancer Center, Pittsburgh, PA, United States of America

**Keywords:** Stereotactic radiosurgery, brain metastasis, memory sparing, memory circuit

## Abstract

**Purpose:**

Radiation dose reduction to major memory circuit substructures (e.g., hippocampus, amygdala, fornix, and corpus callosum) may reduce patients' neurocognitive toxicities. We evaluated memory circuit (MC) dosimetry and determined the feasibility of the “Sparing Memory with Advanced Radiosurgery Targeting” (SMART) technique in patients with brain metastases.

**Materials and methods:**

Retrospective contouring of MC substructures was performed for 101 patients that received single-fraction, linear accelerator (LINAC)-based, monoisocentric SRS plans for increasing numbers of brain metastases: 1, 2–4, 5–9 and ≥ 10. Patients with ≥10 lesions (*n* = 13) were reoptimized using SMART by maximizing dose reduction to MC while maintaining >95% PTV coverage (V100% > 95%). Dosimetric parameters such as D_mean_, D_max_, and D_median_ to MC, cumulative PTV and MC volume were evaluated.

**Results:**

Patients with ≥10 brain metastases received significantly higher D_mean_ (2.9 vs. 0.41 Gy, *p* < 0.0001), D_median_ (2.9 vs. 0.34 Gy, p < 0.0001) and D_max_ (9.0 vs. 1.9 Gy, p < 0.0001) to the MC compared to those with a single metastasis. SMART replanning yielded a significant decrease in D_mean_, D_median_, D_max_, and D_100_ doses to the MC when compared to clinical dosimetry (2.9 vs. 1.8 Gy, *p* = 0.0001; 2.9 vs. 1.8 Gy, *p* = 0.0002; 9.0 vs. 4.7 Gy, *p* = 0.0007; 1.5 vs. 0.98 Gy, *p* = 0.0010, respectively).

**Conclusion:**

Increased number of brain metastases results in higher MC doses. Patients with ≥10 lesions received increased D_mean_/D_median_/D_max_ doses to the MC compared to patients with <10 brain metastases. SMART technique is clinically feasible. Studies evaluating the neurocognitive benefits of SMART are warranted.

## Highlights

• Sparing Memory with Advanced Radiosurgery Targeting (SMART) technique reduces dose to memory circuit (MC) substructures.
• ≥10 brain metastases correlate with higher radiation doses to MC substructures than those with 1, 2-4, and 5-9 metastases.
• SMART is clinically feasible in reducing MC dose in patients with brain metastases, particularly in those with ≥10 lesions.

## 1 Introduction

Brain metastases represent a major oncologic challenge [1]. Because advancements in systemic therapies have improved patient overall survival following a cancer diagnosis, clinicians are increasingly cognizant of late effects/toxicities of cancer therapies in patients with brain metastases [2]. It has been well established that higher radiation doses to certain neuroanatomic structures are associated with cognitive decline, suggesting the necessity of further investigation into more sophisticated radiosurgery techniques [3], [4], [5]. Stereotactic radiosurgery has emerged as a standard of care for treatment of brain metastases, even for patients with multiple lesions [6], [7]. As established by prior studies, treating greater numbers of brain metastases brings to light the question of reducing doses to critical neurocognitive structures (e.g., bilateral hippocampi) to better preserve patient cognition after radiosurgery [4], [8], [9], [10]. The hippocampi remain the most common target of radiation dose avoidance due to its radiosensitivity and its key role in learning, memory, and emotion [4], [11]. However, the benefits of avoiding various collaborative memory circuit substructures (e.g., amygdalae, fornix, and corpus callosum) have yet to be established.

As the number of lesions treated with radiotherapy increases, it also becomes increasingly important to track the dose received by additional structures that control memory and cognition because the hippocampus works as a “circuit” with the amygdalae, corpus callosum, and fornix, all of which have a low propensity for brain metastases [11]. Avoidance of these collaborating substructures, henceforth called the memory circuit (MC), may be more impactful in reducing cognitive deficits than targeting the hippocampus alone.

The goal of this study is to establish baseline dosimetry in treatment plans where MC avoidance was not considered and re-optimize these plans to reduce dose to substructures while maintaining target coverage to determine if MC avoidance in SRS treatment is possible. Specifically, this study replans patients with ≥10 brain metastases using the Sparing Memory with Advanced Radiosurgery Targeting (SMART), a technique developed at our institution, to determine the feasibility of MC avoidant planning in cases of greater number of metastases.

## 2 Methods

### 2.1 Patient selection and criteria

We collected data on a group of consecutively treated patients with SRS for brain metastases at our institution between March 2022 and December 2023. All patients were treated with a single-fraction monoisocentric SRS plan on a linear accelerator (Varian TrueBeam STx). Exclusion criteria included patients with ICD-10 diagnostic codes aside from C79.31, those who received prior intracranial radiation, plans utilizing multiple fractions, multi-isocenter plans, plans with lesions directly on MC substructures, and incomplete treatments. The study was approved by the IRB.

### 2.2 Contouring techniques

**Fig. 1:**
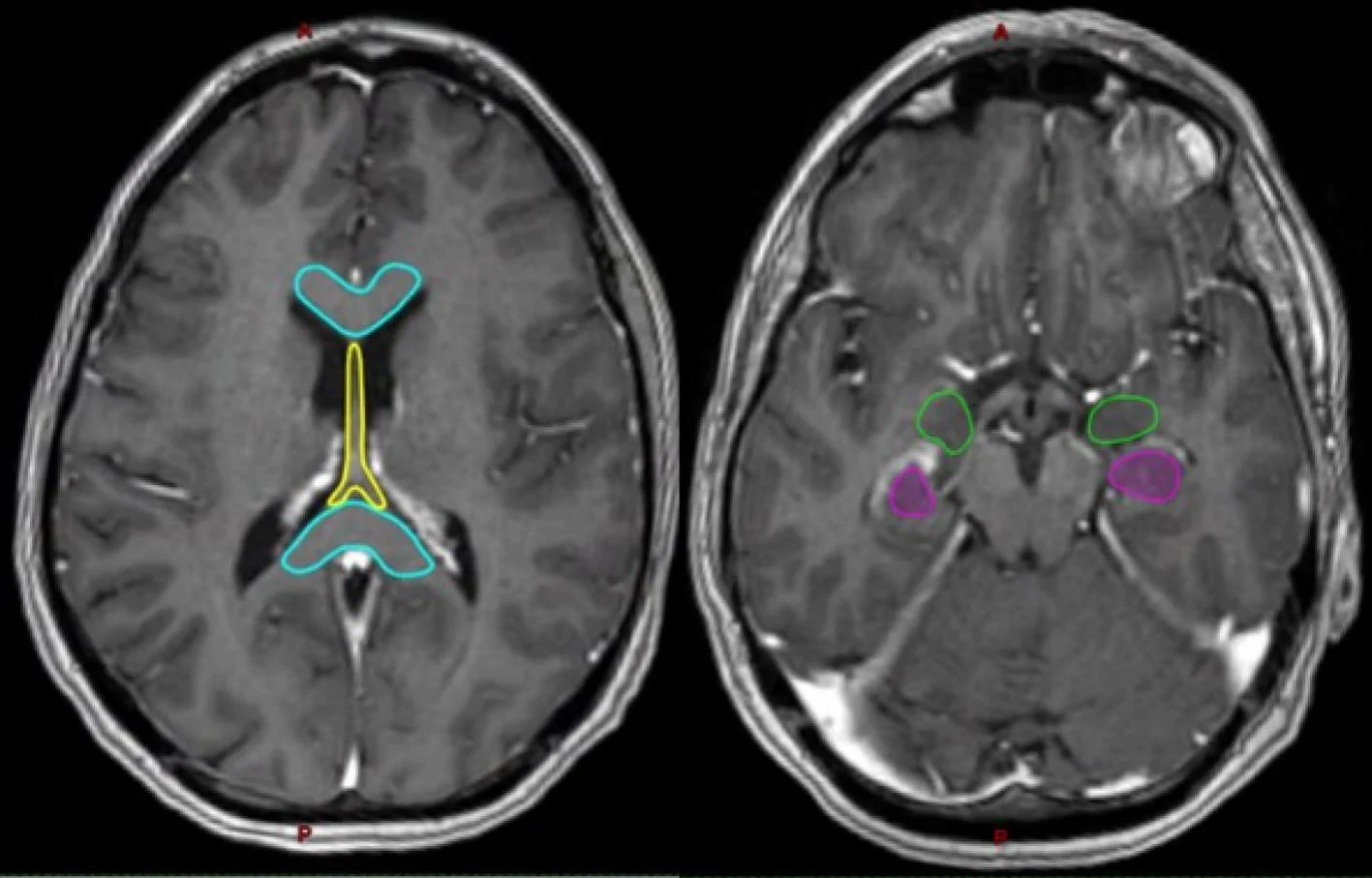
Representative MC contours on MRI brain with contrast which includes corpus callosum (cyan), fornix (yellow), amygdalae (green), and hippocampi (pink). (For interpretation of the references to colour in this figure legend, the reader is referred to the web version of this article.)

Delineation of MC structures followed published contouring atlases [12], [13]. Retrospective manual contouring of patients was performed in Eclipse V16.1.2 (Varian Medical System, part of Siemens Healthineers System, Palo Alto, CA) for the following substructures- left/right hippocampi, left/right amygdalae, fornix, and corpus callosum on axial T1-weighted contrast-enhanced fast spoiled gradient-echo (FSPGR) post-contrast brain MRI **(**Fig. 1**)**. Manual contouring of each patient was performed by research staff and validated for quality assurance by a CNS radiation oncologist, neurosurgeon, and neuroradiologist, with a combined 41 years of experience in CNS structure delineation. Patients were grouped by number of brain lesions (1, 2–4, 5–9, and ≥ 10) for analysis. We created a Boolean sum of all substructures and called it the Memory Circuit (MC). For optimization and for assessing the spatial distributions of metastases with respect to the MC, MC minus PRV (MC-PRV) volumes were created by expanding the MC structure by 2, 5 and 10 mm.

### 2.3 SMART Replanning

SMART replanning was performed only for patients with ≥10 metastases (*n* = 13) by three of our department's medical physicists with a collective experience of 46 years in SRS planning. Optimization and plan preparation were similar to standard SRS plans, giving priority to PTV dose coverage and dose gradient outside the PTV according to institutional guidelines, including limiting the spread of the low-dose (6 Gy) volume and using three ring structures around the PTV with the following start distance for each ring from the PTVs; 0, 5 and 10 mm. Various techniques were used to control the low-dose spread within the normal brain and to prevent any dose bridging through the creation of extra planning structures such as brain minus PTV. In single isocenter, multi-target planning, the location of the isocenter was carefully considered. Even if the TPS was capable of auto-placing the isocenter in the center of the collective volume of the target, the planning physicist assessed the suitability of the location depending on the distribution of targets from each other (clustering of targets, if present), distribution within the brain, and volume of different targets.

All plans were done using Eclipse V16.1.2 and AcurosXB dose calculation algorithm and a 1 mm dose grid matrix. SMART-specific optimization objectives were done as 2nd tier priority and included; limiting maximum dose to 0.03 cc to 6.65Gy or less and limiting the dose to each of the MC substructure to also 6.65Gy. The dose objective of the MC as a whole was given higher priority than the dose objectives of individual substructures. Unless there was an overlap with established organs at risk such as the optic nerves and brain stem, then optimization was geared towards sparing MC substructures.

The MC-PRV volumes were used to control the dose distribution around the MC. A maximum dose objective was used when the PRV volumes do not overlap with PTVs, and a mean dose objective was used when there was an overlap.

SMART planning was done with both 6FFF and 10FFF energies, with 10FFF optimization input being mostly similar to the 6FFF plan. In the Results section and accompanying figures and tables, as well as in the Discussion section reference to SMART planning is understood to be plans with 6FFF modality. For results from 10FFF plans, the 10FFF designation is mentioned explicitly.

### 2.4 Statistical analysis

One-way ANOVA followed by Tukey's multiple comparison tests were performed to compare D_mean_, D_median_, and D_max_ doses to MC between patients with 1, 2–4, 5–9, and ≥ 10 metastases. Linear regression analysis and Pearson correlation coefficient (r) were used to study the correlation between cumulative PTV volume and MC parameters (D_mean_, D_median_, D_max_). Other dosimetric parameters collected include D_mean_, D_median_, and D_max_ to brain minus PTV volume. Volumetric parameters collected include V_5Gy_, V_8Gy_, V_10Gy_, and V_12Gy_ to both the MC and brain minus PTV volume. The Shapiro-Wilk test was performed to compare population distribution between clinical and SMART plans within each dosimetric/volumetric endpoint. Normally distributed endpoint values underwent the paired *t*-test, while the Wilcoxon signed-rank test was performed for non-normally distributed endpoint values.

## 3 Results

**Fig. 2:**
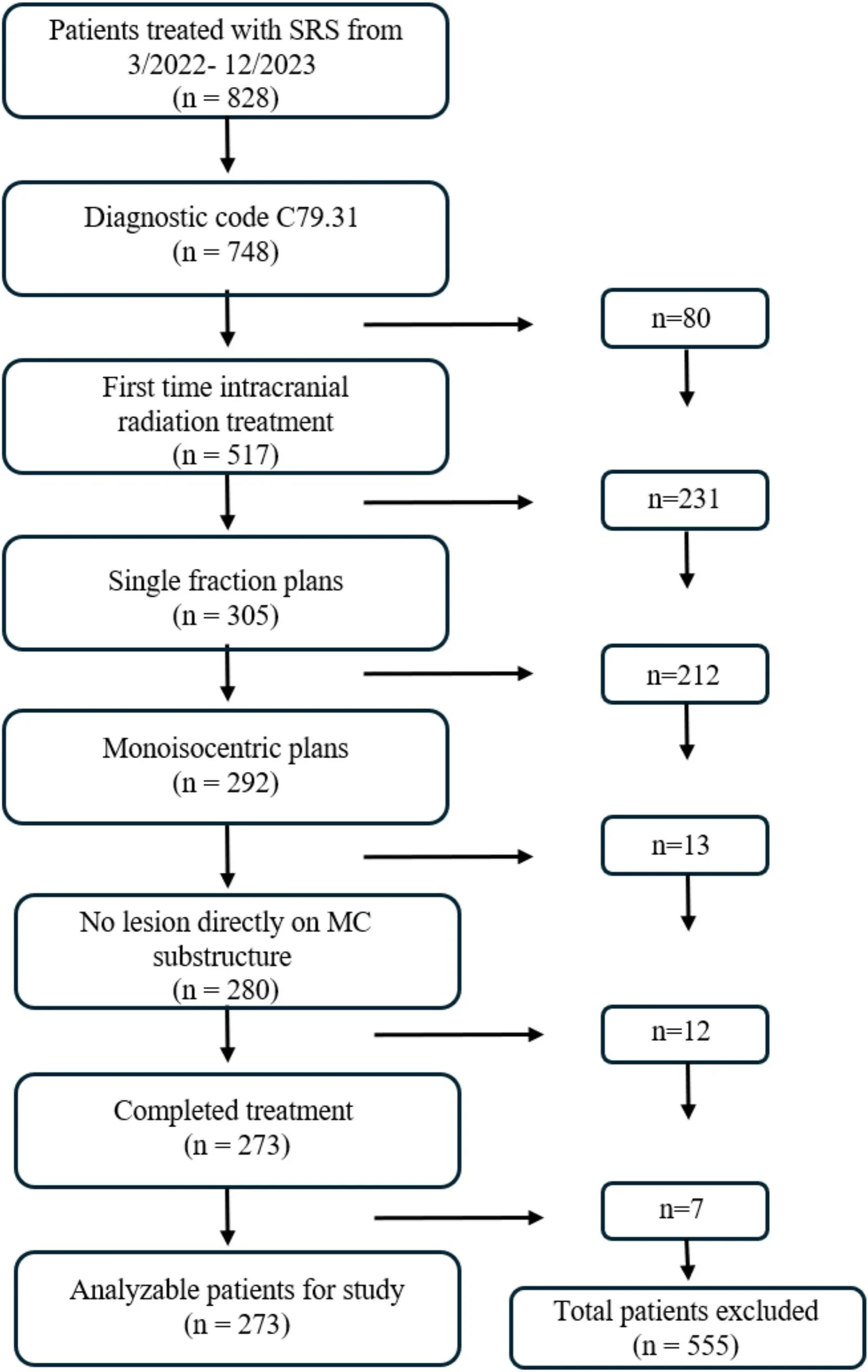
Inclusion criteria for brain metastases analysis.

We identified 828 patients treated with LINAC-based SRS from March 2022 to December 2023 **(**Fig. 2**).** 273 analyzable patients for this study were identified after exclusion factors were applied **(**Fig. 2**)**. From this pool, the first 101 patients receiving SRS treatment for one to ≥10 brain metastases were identified. The average age of patients was 66 years, and brain lesions with a prevalent primary tumor histology of non-small cell lung cancer (41%) **(**Table 1**)**. Patients received a prescription range of 15 to 21 Gy to the PTV with the number of lesions ranging from one to 22. Our institution's standard for PTV margins is 1 mm regardless of number of lesions.

**Table 1:** Patient and Metastases Characteristics.

Characteristics	Patients (*n* = 101)
**Age (y)**	66 (25–89)
**Sex**	**n (%)**
Female	51 (50)
Male	50 (50)
**Histology**	**n (%)**
NSCLC	41 (41)
SCLC	14 (14)
Breast	10 (10)
Renal	5 (5)
Melanoma	13 (13)
Colorectal	3 (3)
Other	15 (15)
**# of brain metastases treated**	**n (%)**
1	30 (30)
2 to 4	30 (30)
5 to 9	28 (28)
10+	13 (13)
**Range of prescription doses (Gy)**	15–21

**Fig. 3:**
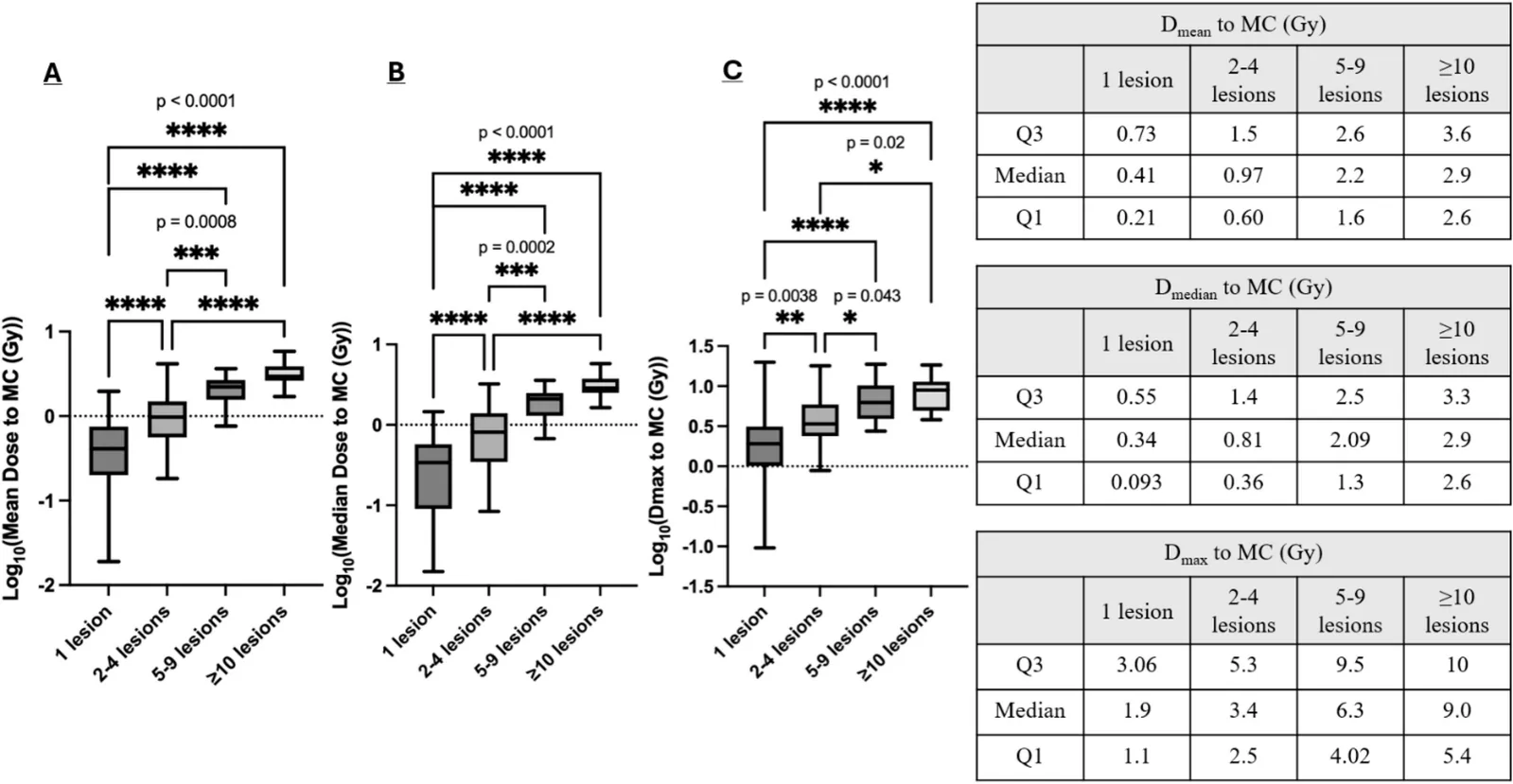
D_mean_ (A)**,** D_median_ (A), and D_max_ (B) doses to MC by increasing numbers of brain metastases. Third quartile, median, and first quartile values calculated.

Compared to patients with one to nine brain metastases, analysis of clinical plans showed patients with ≥10 metastases received significantly greater D_median_, D_mean_, and D_max_ (0.03 cc) doses to MC substructures. Patients with ≥10 lesions received a MC D_mean_ of 2.9 Gy (IQR 2.9–4.3) and single lesion patients received a D_mean_ of 0.41 Gy (IQR 0.21–0.73) **(**Fig. 3A**)**. Patients with ≥10 lesions received an MC D_median_ of 2.9 Gy (IQR 2.6–3.3) and those with one lesion had an MC D_median_ of 0.34 Gy (IQR 0.093–0.55) **(**Fig. 3B**).** While the ≥10 lesion cohort received a D_max_ of 9.0 Gy (IQR 5.4–10) to the MC, patients with one lesion received 1.9 Gy (IQR 1.1–3.06) (*p* < 0.0001) **(**Fig. 3C**).** Increasing cumulative PTV volume suggests a moderate Pearson correlation with D_mean_ **(Supplementary Fig. 1 A)** and D_median_ **(Supplementary Fig. 1B)** to the MC, but a weak correlation with D_max_ to the MC (*r* = 0.5 vs 0.32) **(Supplementary Fig. 1C)**.

**Fig. 4:**
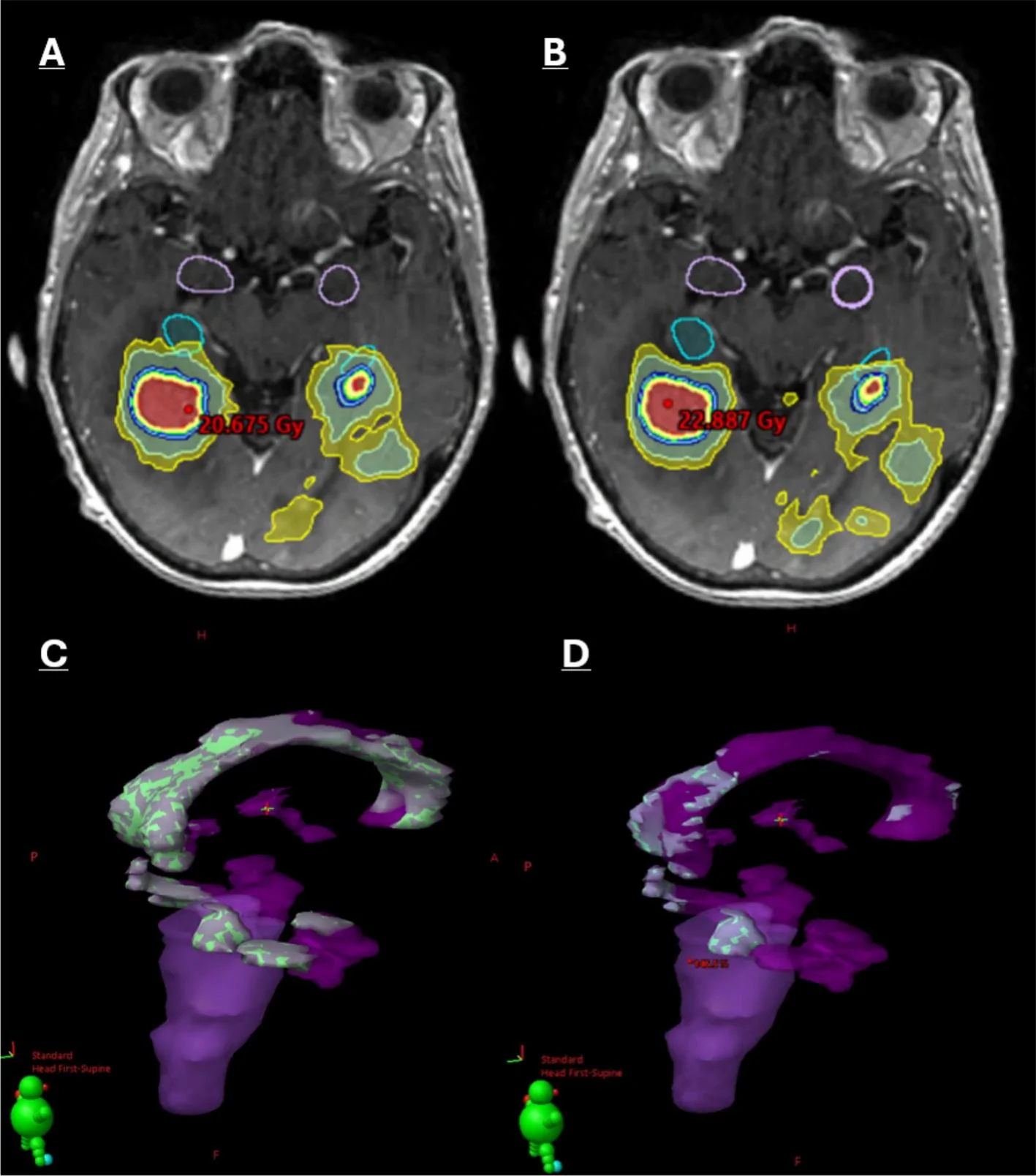
Color-washed FSPGR post-contrast MRI fused axial slices of a patient with several metastases in proximity with the amygdala (lavender) and hippocampi (cyan) in the original treatment plan (A) and SMART replanned treatment (B). Prescription dose was 16 Gy. The 6.65 Gy isodose line is displayed in yellow; this line represents the RTOG 0933 hippocampal dose constraint converted to a biologically effective dose using an α/β of two. 3D visualization of overlap of 4 Gy dose cloud (green) with the MC in the clinical plan (C) and SMART plan (D). (For interpretation of the references to colour in this figure legend, the reader is referred to the web version of this article.)

Based on the significantly higher MC doses seen in patients with ≥10 brain metastases compared to patients with 1, 2–4, and 5–9 brain metastases (Fig. 3), SMART replanning was performed for the subset of patient with ≥10 brain metastases (*n* = 13). SMART replanning showed that MC avoidance was achieved and observed in patients with ≥10 lesions (n = 13) **(**Fig. 4A-B**)**. D_mean_ and D_median_ doses (Gy) to MC structures significantly decreased after SMART replanning (2.9 vs. 1.8, *p* = 0.0001; 2.9 vs. 1.8, *p* = 0.0002) **(**Table 2**)**. D_mean_ to the MC decreased by an average of 6.7% (1.2 Gy) and D_median_ to the MC decreased by an average of 6.3% (1.1 Gy) in SMART replans. Compared to clinical plans, the MC volume receiving 4 Gy in SMART plans was reduced by 6% from 854 cc to 806.5 cc, and the MC overlapped volume was reduced by 85% from 11.4 cc to 1.8 cc **(**Fig. 4C-D**)**. While MC structures in clinical plans received a median D_max_ of 9.0 Gy, SMART plans achieved a significantly decreased 4.7 Gy (*p* = 0.0007) **(**Table 2**)**. A significant decrease in D_100_ to MC was also observed (1.5 vs. 0.98, *p* = 0.001) **(**Table 2**)**. SMART plans did not significantly differ in PTV coverage. A PTV coverage of >95% was maintained when patients underwent replanning, with a median D_max_ to PTV of 23 Gy in clinical plans and 24 Gy in SMART plans (*p* = 0.85) **(**Table 2**)**. Median D_95%,_ D_98%_, and D_mean_ to the PTV was unchanged between clinical plans and SMART plans (19 vs. 19 Gy, *p* = 0.77; 18 vs. 18 Gy, *p* = 0.76; 21 vs. 21 Gy, *p* = 0.74) **(**Table 2**).** Median V_5Gy_ and V_8Gy_ to the MC decreased significantly (0.80 vs. 0.0048 cc, *p* = 0.037; 0.18 vs. 0.11 cc, *p* = 0.0078), while V_10Gy_ and V_12Gy_ increased (0.012 vs. 0.11 cc, *p* = 0.031; 0.043 vs. 0.13 cc, *p* = 0.13) **(**Table 2**)**. D_median_ dose to brain minus PTV volume shows a significant decrease (2.8 vs. 2.7 Gy, *p* = 0.018) **(**Table 2**).** V_5Gy_ to V_12Gy_ to the brain minus PTV volume showed nonsignificant changes between plans **(**Table 2**)**.

**Table 2:** Dosimetric outcomes for SMART replanning.

Dosimetric end points	Clinical plan (n = 13)		SMART replanned (n = 13)		*p*-value
	Median	IQR	Median	IQR	
D_mean_ to MC (Gy)	2.9	2.6–3.2	1.8	1.7–2.6	0.00010^a^
D_median_ to MC (Gy)	2.9	2.6–3.3	1.8	1.7–2.4	0.00020^a^
D_max_ to MC (Gy)	9.0	5.4–10	4.7	3.6–7.5	0.00070^a^
D_100_ to MC (Gy)	1.5	1.3–1.5	0.98	0.82–1.4	0.0010^a^
V_5Gy_ to MC (cc)	0.80	0.038–2.2	0.0048	0–0.4	0.037^b^
V_8Gy_ to MC (cc)	0.18	0–0.32	0.11	0–0.0022	0.0078^b^
V_10Gy_ to MC (cc)	0.012	0–0.010	0.11	0–0	0.031^b^
V_12Gy_ to MC (cc)	0.043	0–0.00011	0.13	0–0	0.13^b^
D_max_ to PTV (Gy)	23	20–25	24	23–25	0.085^a^
D_95%_ to PTV (Gy)	19	17–19	19	17–19	0.77^a^
D_98%_ to PTV (Gy)	18	16–19	18	16–18	0.76^a^
D_mean_ to PTV (Gy)	21	18–21	21	19–21	0.74^a^
Dose to Brain-PTV (Gy)	2.8	2.5–3.3	2.7	2.2–3.2	0.018^a^
V_5Gy_ to Brain-PTV (cc)	168	133–228	121	85–193	0.055^a^
V_8Gy_ to Brain-PTV (cc)	51	40–69	41	31–65	0.094^a^
V_10Gy_ to Brain-PTV (cc)	29	23–44	26	19–39	0.099^a^
V_12Gy_ to Brain-PTV (cc)	18	12–27	16	12–25	0.10^a^

Paired *t*-test (a) performed on values that passed the Shapiro-Wilk test. Wilcoxon signed-rank test (b) performed on non-normally distributed values.

D_mean_, D_median_, and D_100_ doses to the brain minus PTV volume decreased significantly in SMART replans **(Supplementary Fig. 3)**. D_max_ to the OARs such as the brainstem and optic apparatus did not change significantly after SMART replanning **(Supplementary Table 1).** Average D_40%_ of MC structures in the SMART plans were significantly lower than original plans (3.3 vs. 2.1 Gy, *p* = 0.0042) **(Supplementary Fig. 4).** Dosimetric end points were compared between SMART plans using 6-FFF and 10-FFF beams. Mean D_40%_ to both the MC and brain minus PTV volume was nonsignificant between 6 and 10-FFF plans **(Supplementary Fig. 5 A-B).** Average D_mean_, D_median_, and D_max_ to the MC were significantly higher in 10-FFF plans (2.1 vs. 2.4 Gy, *p* < 0.0001; 2.0 vs. 2.2 Gy, p < 0.0001; 6.8 vs. 7.0 Gy, p < 0.0001) **(Supplementary Fig. 6 A-B).** Similarly, D_mean_ and D_median_ to the brain minus PTV volume were significantly higher in 10-FFF plans (p < 0.0001) **(Supplementary Fig. 6D-E).** Representative contours of segmented MC substructures on an axial plane **(Supplementary Fig. 7)**, coronal plane **(Supplementary Fig. 8)**, and sagittal plane **(Supplementary Fig. 9)** were captured.

## 4 Discussion

SRS remains standard of care for limited volume/number brain metastases, and its scope of utilization continues to grow as the rapid advancement of imaging techniques and treatment planning continue to increase the quality of life and survival of patients [6], [7], [8], [14]. In this study, LINAC-based monoisocentric SRS plans were retrospectively contoured to determine the feasibility of MC-sparing SRS in a sample group where dosimetry to MC structures was not considered. Patients treated for ≥10 brain metastases received increased D_mean_, D_median_, and D_max_ to the MC compared to patients with <10 brain metastases. As the number of lesions treated with SRS increases, the dosimetric sparing of memory circuit substructures will become clinically important in the near future [7], [8], [15].

SMART replanning yielded significantly reduced MC doses in all patients with ≥10 brain metastases while maintaining appropriate PTV coverage. In addition, replanning resulted in a significant decrease in doses to the brain minus PTV volume. Birer et al. conducted similar dosimetric comparisons for bilateral hippocampi between original and re-optimized plans and reported an average reduction of 27% (1.36 Gy) in mean dose to bilateral hippocampi and 39% (3.82 Gy) in maximum dose [16]. Memory circuit avoidance has been investigated in the context of WBRT in a prior phase II clinical trial investigating patients with >15 lesions [17]. Further data on radiation dose constraints to the memory circuit beyond the hippocampus is needed.

Emerging research shows that lower radiation doses can lead to cognitive, cellular, and molecular changes [18], [19], [20], [21]. A D_40%_ > 2.5 Gy to hippocampi correlates with significant decline in verbal learning and memory retention [22]. In our study, D_40%_ of MC was collected for patients with greater than 10 lesions and yielded an average of >2.5 Gy in both original and SMART plans. V_12Gy_ is a widely established parameter for predicting the risk of radiation necrosis (RN) for SRS [23]. Doses as low as 100 mGy can lead to decreased hippocampal neurogenesis and morphological changes in neuronal cells [18]. Blonigen and colleagues found that V_8Gy_, V_10Gy_, V_12Gy_, V_14Gy_, and V_16Gy_ show significant correlation with symptomatic RN after SRS treatment [24]. Another study found that doses as low as V_5Gy_ are capable of increasing spontaneous neuronal network activity with the potential to disrupt cognitive function [25]. As patients with brain metastases are living longer and may undergo increased number of brain radiotherapy courses, it is important to critically examine the impact of low radiation dose spillage to the healthy brain tissue.

As the radiotherapy field progresses towards sparing a multitude of structures in cases of increasing numbers and volumes of lesions, further discussion on the emergence of monoisocentric, LINAC-based SRS to achieve sharp dose gradients and reduce normal brain injury is warranted. This technique is increasingly more common in clinical practices and studies suggest its efficiency in speed and local control while treating multiple brain metastases compared to traditional multi-isocenter SRS workflows [26], [27], [28], [29], [30], [31], [32], [33]. Recent publications have looked into the dosimetry of multi-lesion single isocenter plans focusing on low-dose spread in normal brain tissue, treating the brain as one single organ [34], [35]. Monoisocentric LINAC-based SRS is an efficient technique for multiple brain lesions due to the speed of treatment and highly conformal dose distribution from the multileaf collimator [36], [37]. Despite these benefits, the technique is associated with increased low dose spillage to the brain minus PTV volume [38]. The major cause is twofold- first, the interleaf spaces in the collimator are susceptible to increased low dose seepage and second, the island blocking issue where multiple lesions share the same leaf pairs, creating an unshielded area of healthy brain tissue [39], [40]. Low dose spread to memory-related structures could have cellular and cognitive effects that differ from spillage to nonrelated white matter.

Incorporating the MC as an avoidance structure in planning may be an effective strategy to improve neurocognitive outcomes in patients undergoing monoisocentric LINAC-based SRS. The impact of increased doses on individual MC structures is well documented. An investigation into radiotherapy-induced white matter injury to memory-related structures found that dose-dependent injury to the fornix and parahippocampal cingulum (PHC) correlates with decreased visuospatial memory [41]. Higher radiation doses to bilateral hippocampi are associated with significant decline in verbal learning and recall [5], [42], [43]. Radiotherapy-induced atrophy of the left amygdala leads to significant decline in verbal memory, while volume loss in the right amygdala correlates with anxiety symptoms [44]. The structural integrity connecting individual memory-related structures also raises questions as to how injury to one mediates the function of another. Injury to the fornix leading to a loss of structural connection to the hippocampi is a significant indicator for hippocampal atrophy [45]. The loss of white matter volume in the fornix alone correlates with cognitive deterioration in healthy elderly individuals [46]. Injury to the adjacent corpus callosum is a predictor of declining attention and processing speed after radiotherapy [47]. Given the cognitive decline followed by injury to individual MC structures, determining the radiosensitivity of each merits further research. Collectively, these memory structures make up an established circuit where localized damage is a predictor of memory loss [48].

With the completion of this study, we will establish a longitudinal clinical framework that facilitates the identification of novel organs at risk for SRS planning and measures the sensitivity of functional brain regions to radiation dose. The current study has several limitations. This is a single-institution retrospective study, which makes the data susceptible to bias and confounding variables. The ≥10 lesion cohort analyzed for SMART replanning consists of a small sample size (*n* = 13), making it vulnerable to random variation. Another weakness is the reliance on hippocampal constraints used in RTOG 0933 as a reference when measuring MC dosimetry. At this time, there are no known constraints specific to MC substructures established in the field. One notable strength of this study is its novelty in analyzing MC dosimetry in SRS plans, when previous studies solely focused on application in WBRT. By replanning patients with ≥10 lesions, we established a baseline dosimetric and contouring framework for applying MC avoidance in patients with greater numbers of metastases.

## 5 Conclusion

In summary, this study shows that patients with increasing number of brain metastases receive higher doses to memory-critical substructures in single fraction monoisocentric LINAC-based SRS plans. Our results demonstrate that delivering the SMART planning technique by sparing neurocognitive structures beyond the hippocampi (including amygdalae, corpus callosum, and fornix) is achievable. In all cases with ≥10 brain metastases, it was clinically feasible to generate an MC-sparing plan without sacrificing target coverage. Future evaluations of the neurocognitive effects of the SMART technique are warranted.

## CRediT authorship contribution statement

**Nina Lo:** Writing – review & editing, Writing – original draft, Project administration, Methodology, Investigation, Formal analysis, Data curation. **Shada Wadi-Ramahi:** Writing – review & editing, Visualization, Validation, Supervision, Software, Conceptualization. **Hong Wang:** Writing – review & editing, Validation, Supervision, Data curation. **Doris Dimitriadou:** Writing – review & editing, Visualization, Validation, Supervision, Software, Project administration, Methodology. **Ron Lalonde:** Writing – review & editing, Visualization, Validation, Supervision, Software, Methodology. **Travis McCaw:** Writing – review & editing, Visualization, Validation, Supervision, Software, Methodology. **Kanika Sarna:** Writing – review & editing. **Christopher Wilke:** Writing – review & editing, Visualization, Validation, Supervision. **Joseph Mettenburg:** Writing – review & editing, Visualization, Validation, Supervision. **Kalil Abdullah:** Writing – review & editing, Visualization, Validation, Supervision. **Andrew H. Zureick:** Writing – review & editing, Visualization, Validation, Supervision. **Serah Choi:** Writing – review & editing, Visualization, Validation, Supervision, Software, Resources, Project administration, Methodology, Funding acquisition, Conceptualization.

## Declaration of competing interest

The authors declare the following financial interests/personal relationships which may be considered as potential competing interests: Serah Choi reports a relationship with Telix Pharmaceuticals Limited that includes: consulting or advisory. Serah Choi reports a relationship with GT Medical Technologies Inc that includes: consulting or advisory. If there are other authors, they declare that they have no known competing financial interests or personal relationships that could have appeared to influence the work reported in this paper.
