# Supplementary material for: Memory circuit dosimetry in patients with brain metastases treated with Sparing Memory with Advanced Radiosurgery Targeting (SMART) technique
Source: Clin Transl Radiat Oncol. 2026 Aug 15;61:101267. doi: 10.1016/j.ctro.2026.101267 (PMC13524723; doi:10.1016/j.ctro.2026.101267)
Supplement: Supplementary file 1 [file mmc1.docx]

**SUPPLEMENTARY DATA**

**Supplementary Figure 1.** Linear regression analysis and the Pearson correlation (r) of cumulative PTV volume with D_mean_ (A), D_median_ (B), and D_max_ (C) received by the memory circuit (MC), where R^2^ indicates the percentage of variation in the outcome variable that is explained by cumulative PTV volume. Trendline represents regression line fit.

**Supplementary Figure 2.** Comparison of D_mean_ (A), D_median_ (B), D_max_ (C), and D_100_ (D) doses to the MC between clinical plans and SMART replans in box and whisker plot form.

**Supplementary Figure 3.** Comparison of D_mean_ (A), D_median_ (B), D_max_ (C), and D_100_ (D) doses to the normal brain volume between clinical plans and SMART replans in box and whisker plot form.

**Supplementary Figure 4.** Mean D_40%_ delivered to MC in the clinical plans compared to the SMART replans.

**Supplementary Figure 5.** D_40%_ delivered to MC (A) and normal brain volume (B) between 6FFF and 10FFF energy SMART replans.

**Supplementary Figure 6**. D_Mean_, D_median_, and Dmax doses to the MC (A-C) and normal brain volume (D-F) compared between 6FFF and 10FFF energy SMART replans.

**Supplementary Figure 7.** Representative view of the hippocampi (lavender), amygdalae (pink), corpus callosum (cyan), and fornix (yellow) contoured on an axial plane.

**Supplementary Figure 8.** Representative view of the hippocampi (lavender), amygdalae (pink), corpus callosum (cyan), and fornix (yellow) contoured on a coronal plane.

**Supplementary Figure 9.** Representative view of the hippocampi (lavender), amygdalae (pink), corpus callosum (cyan), and fornix (yellow) contoured on a sagittal plane.

**Supplementary Table 1. Dosimetric outcomes for organs at risk D_max_ in clinical plans and SMART replans**

| **Dosimetric end points** | **Clinical plan (n=13)** | | **SMART replan (n=13)** | | ***p*-value** |
| --- | --- | --- | --- | --- | --- |
|  | **Median** | **IQR** | **Median** | **IQR** |  |
| D_max_ to Brainstem (Gy) | 5.1 | 4.1-7.8 | 5.1 | 3.6-6.1 | 0.73^b^ |
| D_max_ to Optic Chiasm (Gy) | 3.8 | 3.0-4.4 | 2.9 | 2.4-4.1 | 0.29^b^ |
| D_max_ to Optic Nerve L (Gy) | 2.3 | 1.7-3.3 | 2.6 | 2.0-3.3 | 0.16^a^ |
| D_max_ to Optic Nerve R (Gy) | 3.1 | 2.1-3.6 | 3.2 | 1.8-3.6 | 0.44^a^ |

Paired *t*-test (a) performed on values that passed the Shapiro-Wilk test. Wilcoxon signed-rank test (b) performed on non-normally distributed values.
